# Polygenic Profiles Are Associated with Multidomain Biochemical Adaptations Across a Competitive Season in Professional Football Players: A Longitudinal Observational Study

**DOI:** 10.3390/genes17080927

**Published:** 2026-08-07

**Authors:** Jorge Carretero-García, David Varillas-Delgado

**Affiliations:** Exercise and Sport Science, Faculty of Health Sciences, Universidad Francisco de Vitoria, 28223 Madrid, Spain; jorge.carretero@ufv.es

**Keywords:** polygenic score (TGS), sports genomics, biochemical biomarkers, elite football players, metabolic efficiency, precision sports medicine

## Abstract

**Background/Objectives**: The physiological adaptations required to sustain elite football performance are influenced by both genetic background and dynamic biochemical responses, although their interaction across a full competitive season remains insufficiently characterized. This study aimed to examine the association between polygenic profiles and longitudinal biochemical adaptations in professional football players. **Methods**: Forty male professional football players competing in the Spanish league were monitored across two consecutive seasons. Blood samples were collected at six time points representing different phases of the competitive cycle. Biomarkers related to muscle metabolism, iron status, and hepatic function were analyzed. Polygenic profiles were calculated using Total Genotype Scores (TGS) for muscle performance, hepatic resilience, and metabolic efficiency. Associations were initially explored using Pearson correlations and subsequently evaluated using linear mixed-effects models accounting for repeated measurements within subjects. **Results**: Exploratory correlation analyses identified several associations between polygenic profiles and biochemical markers. Muscle performance TGS was inversely associated with serum iron (r = −0.36, *p* = 0.017) and positively associated with CK (r = 0.32, *p* = 0.041), Hb (r = 0.29, *p* = 0.046), and Hct (r = 0.33, *p* = 0.024). Hepatic resilience TGS showed inverse associations with ALT (r = −0.39, *p* = 0.012), urea (r = −0.51, *p* = 0.011), and BUN (r = −0.51, *p* = 0.011). Metabolic efficiency TGS was negatively associated with AST (r = −0.43, *p* = 0.044), ALT (r = −0.33, *p* = 0.025), and GGT across multiple time points (*p* = 0.001–0.013). However, although several nominal associations emerged in linear mixed-effects models accounting for repeated measurements, none remained statistically significant after false discovery rate correction. These findings should therefore be interpreted as exploratory and hypothesis-generating. **Conclusions**: Polygenic profiles may be associated with inter-individual variability in biochemical adaptations throughout a competitive season. These findings suggest the integration of genomic and biochemical data in precision athlete monitoring, while highlighting causal relationships and predictive applications require further investigation.

## 1. Introduction

Elite athletes must sustain peak performance across prolonged competitive seasons, a capacity shaped by complex interactions among physiological, environmental, and individual factors. Recent evidence underscores that performance variability is influenced not only by training and tactical preparation but also by intrinsic biological characteristics and adaptive responses to cumulative load [1,2]. In professional football, maintaining high-level performance requires integrated approaches that combine physical conditioning, recovery strategies, and continuous monitoring of physiological markers [1,3,4]. Emerging frameworks advocate precision-based methodologies, where individualized interventions in training and nutrition aim to optimize performance while minimizing injury risk [4,5,6].

Central to these strategies, blood biomarkers have become essential tools in sports medicine, providing objective insights into training load, recovery status, physiological adaptation, and early signs of fatigue or injury risk in elite athletes [3,7,8]. Biomarker concentrations fluctuate throughout the competitive cycle—preseason, in-season, and transition periods—reflecting the dynamic nature of training stress and recovery demands [9,10]. The systematic integration of biochemical biomarkers into athlete monitoring frameworks has been shown to enhance decision-making processes and support evidence-based interventions aimed at optimizing performance while preserving athlete health [11,12]. Beyond global physiological assessment, specific blood-derived markers provide actionable information on key physiological domains, including muscle integrity, oxygen transport capacity, metabolic resilience, and recovery status [12,13]. In this context, hematological indicators such as iron-related parameters and hemoglobin play a critical role in determining aerobic capacity and oxygen delivery, both of which are essential for endurance performance [14,15,16]. Alterations in iron status have been consistently associated with impaired physical performance, particularly in athletes exposed to high training loads or endurance demands [14]. Furthermore, enzymatic markers such as creatine kinase (CK) and lactate dehydrogenase (LDH) are widely used to reflect muscle damage, training load, and adaptive responses to exercise-induced stress [13,17,18]. Fluctuations in these biomarkers provide valuable insight into the balance between workload and recovery, as well as the athlete’s capacity to adapt to repeated physiological stress [19]. The dynamic fluctuations of these biomarkers across the competitive season provide a multidimensional perspective on performance, fatigue, and physiological adaptation, supporting the development of individualized strategies to maintain health and optimize athletic output [20,21,22]. Importantly, these biochemical signals do not operate in isolation, as their variability is partially influenced by genetic factors that modulate metabolic efficiency, recovery capacity, and susceptibility to physiological stress [21,22].

In this context, the integration of biomarker profiling with genomic information has been proposed as a promising approach for advancing precision athlete monitoring. Such an approach enables the design of personalized training, nutrition, and recovery strategies that are aligned with each athlete’s unique biological and genetic architecture, thereby enhancing both performance optimization and long-term athlete health [22,23,24]. Athletic performance is a complex polygenic trait influenced by multiple genetic variants that collectively modulate muscle structure, metabolic efficiency, and recovery capacity. Among the most extensively studied polymorphisms, variants in the angiotensin-converting enzyme (*ACE* I/D; rs4646994) and alpha-actinin-3 (*ACTN3* c.1729C>T; rs1815739) genes have been consistently associated with endurance- and power-oriented phenotypes, respectively [25,26]. Beyond these classical markers, additional polymorphisms in genes such as adenosine monophosphate deaminase 1 (*AMPD1* c.34C>T; rs17602729), creatine kinase muscle-type (*CKM* c.*800A>G; rs8111989), and myosin light chain kinase (*MLCK*; rs2700352 and rs28497577) contribute to inter-individual variability in muscle metabolism and contractile adaptation to exercise [27,28,29]. Furthermore, genetic variants involved in oxidative stress regulation and detoxification pathways, as cytochrome P450 2D6 (*CYP2D6* c.506-1G>A; rs3892097), glutathione S-transferase Pi 1 (*GSTP* c.313A>G; rs1695), glutathione S transferase Mu 1 (*GSTM1* deletion/null variant) and glutathione S transferase Theta 1 (*GSTT* deletion/null variant) have been associated with differential responses to training stress and recovery processes under high workloads [9,30]. Variants in genes related to iron metabolism and mitochondrial biogenesis, such as the Homeostatic Iron Regulator (*HFE*; rs1799945), *AMPD1*, and peroxisome proliferator-activated receptor gamma coactivator-1 alpha (*PGC1α*; rs8192678), further influence aerobic efficiency and energy production capacity [1,31]. Importantly, these genetic factors do not act independently; rather, they interact within a polygenic framework, where the combined effect of multiple variants contributes to the overall athletic phenotype and response to training stimuli [9,32].

The Total Genotype Score (TGS) has been proposed as a methodological approach to estimate the cumulative influence of multiple genetic variants on complex athletic traits [9,33]. By integrating the effects of several functional polymorphisms, TGS provides a more comprehensive representation of an individual’s genetic predisposition compared to single-gene analyses, which often fail to capture the multifactorial nature of performance [34,35]. Previous research has suggested that elite athletes tend to present distinct polygenic profiles compared to non-athletic populations, potentially favoring physiological traits such as improved metabolic efficiency, enhanced oxygen transport, and greater resilience to exercise-induced stress [9,36]. However, it is important to note that the predictive capacity of these models remains limited, reinforcing the need for integrative and multi-layered approaches.

Despite growing interest in precision sports medicine, few studies have simultaneously examined polygenic profiles and repeated biochemical assessments across an entire competitive season in elite football players. Understanding how genetic predisposition relates to longitudinal physiological variability may provide new insights into athlete adaptation under real-world competitive conditions. In this context, the combination of genomic profiling with longitudinal biomarker monitoring offers a promising framework for precision athlete management. This integrative strategy facilitates the development of individualized training, nutrition, and recovery interventions tailored to each athlete’s unique biological and genetic profile, with the potential to optimize performance while minimizing health risks [37,38,39].

Therefore, the aim of this study was to investigate the influence of athletes’ genetic predispositions on their physiological responses in professional football players by identifying associations between specific genetic profiles and key biochemical variables across a full competitive season. We hypothesized that professional football players with more favorable polygenic profiles would exhibit different biochemical adaptation patterns throughout the competitive season, particularly in biomarkers related to muscle metabolism, iron homeostasis, hepatic function, and metabolic regulation.

## 2. Materials and Methods

### 2.1. Participants

This longitudinal observational study included 40 male professional football players registered with a club competing in the Spanish professional football league. Participants were followed prospectively throughout two consecutive competitive seasons (2020–2021 and 2021–2022). Baseline demographic and anthropometric characteristics are presented in Table 1.

The eligibility criteria for this study included: (i) male professional football players aged ≥ 18 years; (ii) being part of the first-team squad and actively involved in team activities for at least six months during the competitive season; (iii) regular participation in training sessions during the six months preceding study inclusion. Players were excluded if they had sustained traumatic injuries resulting in absence from training sessions or match participation during the six months prior to the beginning of the study.

All participants provided written informed consent before enrolment. The study protocol was approved by the Research Ethics Committee of Francisco de Vitoria University (IRB UFV 32–2020) and was conducted in accordance with the Declaration of Helsinki for research involving human participants (1964; revised in 2013).

### 2.2. DNA Sample Collection and Genotyping

Biological samples were collected from buccal epithelial cells using SARSTEDT buccal swabs and stored at 4 °C until genetic analysis. Genomic DNA was extracted at VIVOLabs (Madrid, Spain) using the automated QIAcube system (QIAGEN, Venlo, The Netherlands). DNA concentration ranged between 25 and 40 ng/µL, and extracted samples were stored in 100 µL aliquots at −20 °C until genotyping.

The polymorphisms included in the genetic profile related to muscle performance were *ACE* I/D (rs4646994), *ACTN3* c.1729C>T (rs1815739), *AMPD1* c.34C>T (rs17602729), *CKM* c.800A>G (rs8111989), *MLCK* c.49C>T (rs2700352), and *MLCK* c.37885C>A (rs28497577). The genetic profile related to hepatic metabolism included *CYP2D6* c.506-1G>A (rs3892097), *GSTP1* c.313A>G (rs1695), *GSTM1* deletion/null variant, and *GSTT1* deletion/null variant. Finally, the metabolic efficiency profile comprised *HFE* c.187C>G (rs1799945), *HFE* c.845G>A (rs1800562), *AMPD1* c.34C>T (rs17602729), and *PGC1α* c.1444G>A (rs8192678). Genotyping was conducted using single-nucleotide primer extension (SNPE) with the SNaPshot Multiplex Kit (Thermo Fisher Scientific, Waltham, MA, USA), and results were analyzed via capillary electrophoresis on an ABI3500 instrument (Applied Biosystems, Foster City, CA, USA). The bioinformatic interpretation was performed using GeneMapper 5.0 software (Applied Biosystems, Foster City, CA, USA).

### 2.3. Blood Sampling and Biochemical Analyses

Blood samples were collected at six predefined time points throughout the two competitive seasons: (1st), pre-season; (2nd), beginning of the season; (3rd), first third of the season; (4th), mid-season; (5th), second half of the season; (6th), end of the season. These points were selected to capture potential variations in biochemical markers across different phases of the competitive cycle.

All samples were obtained at 8:00 a.m. following a 10–12 h overnight fast, with participants in a seated resting position. A total volume of 10 mL of venous blood was collected from the antecubital vein. Blood samples were collected into ethylenediaminetetraacetic acid (EDTA) tubes for hematological analyses, while additional samples were collected into serum-separating tubes for biochemical determinations.

The association between genetic profiles and biochemical parameters was evaluated by analyzing a set of biomarkers related to muscle performance, hepatic function, and metabolic efficiency. The muscle performance-related biomarkers included ferritin (Ft), serum iron (Fe), hemoglobin (Hb), hematocrit (Hct), creatine kinase (CK), lactate dehydrogenase (LDH), creatinine, urea, and blood urea nitrogen (BUN). The hepatic function-related profile included aspartate aminotransferase (AST) and alanine aminotransferase (ALT), urea, LDH, creatinine, and uric acid. Finally, metabolic efficiency-related biomarkers included CK, LDH, AST, ALT, GGT, Fe, Ft, transferrin, Hct, Hb, mean corpuscular volume (MCV), mean corpuscular hemoglobin (MCH), and mean corpuscular hemoglobin concentration (MCHC).

### 2.4. Polygenic Profiles of Selected Genes

For each genetic profile, a Total Genotype Score (TGS) was calculated to estimate the combined effect of the selected polymorphisms, following the methodology described by Williams and Folland [35] (Table 2).

Because robust and externally validated effect sizes are not currently available for the selected polymorphisms in relation to the sport-specific phenotypes investigated in professional football players, all variants were assigned equal weights following the original TGS methodology proposed by Williams and Folland [35]. This approach has been widely applied in sports genomics studies evaluating polygenic profiles in elite athletes and assumes that the cumulative contribution of multiple biologically relevant variants may be more informative than isolated single-gene effects [9,28]. Although equal weighting does not account for potentially different effect magnitudes among polymorphisms, it avoids introducing uncertain weighting coefficients derived from heterogeneous populations, phenotypes, or study designs. Therefore, the present TGS should be interpreted as a hypothesis-driven composite genetic profile rather than as a genome-wide polygenic score based on GWAS-derived effect estimates.

Each genotype was assigned a score based on its presumed functional relevance in relation to performance-related phenotypes in professional football players, as previously reported. Specifically, genotypes classified as “optimal” were awarded 2 points, heterozygous genotypes were assigned 1 point, and genotypes considered “less favorable” were given 0 points. This scoring system was established according to prior evidence linking specific allelic variants to beneficial or detrimental effects on performance-related traits.

The individual scores for each polymorphism were subsequently summed to obtain a composite score representing the theoretical maximum genetically favorable profile (perfect TGS), assuming the presence of all optimal genotypes.

To facilitate interpretation and comparability across individuals and profiles, the raw TGS was normalized onto a 0–100 arbitrary unit (a.u.) scale using the following formula:

TGS_muscle performance_ = (GS*_ACTN_*_rs1815739_ + GS*_ACE_*_rs4646994_ + GS*_AMPD1_*_rs17602729_ + GS*_CKM_*_rs8111989_ + GS*_MLCK_*_rs2700352_ + GS*_MLCK_*_rs28497577_) × 100/12

TGS_hepatic resilience_ = (GS*_CYP2D6_*_rs3892097_ + GS_*GSTP1*_
_rs1695_ + GS*_GSTM_* + GS*_GSTT_*) × 100/8

TGS_metabolic efficiency_ = (GS*_HFE_*_rs1799945_ + GS*_HFE_*_rs1800562_ + GS*_AMPD1_*_rs17602729_ + GS*_PGC1α_*_rs8192678_) × 100/8

This transformation allowed for a standardized quantitative assessment of the polygenic contribution to the phenotype of interest, with higher scores indicating a greater proportion of favorable alleles within a given profile.

### 2.5. Statistical Analysis

Statistical analyses were performed using R (R Foundation for Statistical Computing, Vienna, Austria) implemented through RStudio 4.6.1 (Posit Software, Boston, MA, USA). Data were imported from .xlsx files, checked for duplicates and missing values, and reshaped into long format (one row per subject, time point, TGS profile, and biomarker).

Hardy–Weinberg equilibrium (HWE) was assessed for all SNPs using exact tests based on observed and expected genotype frequencies [40]. Variants deviating from HWE (*p* < 0.050) were retained because the study population consisted of a highly selected cohort of professional football players rather than a representative population sample. Consequently, HWE deviations were interpreted cautiously. Variants showing no allelic variation within the study population (monomorphic loci) were excluded from HWE testing because genotype frequency comparisons are mathematically uninformative under complete absence of variability.

Normality was evaluated using the Shapiro–Wilk test and homogeneity of variances using Levene’s test. Outliers were identified for each biomarker–time point combination using the IQR criterion (below Q1 − 1.5 × IQR or above Q3 + 1.5 × IQR). Two analytical pipelines were performed in parallel: a primary analysis including all observations and a sensitivity analysis excluding dynamically identified outliers. Because outlier detection was conducted separately for each biomarker, time point, and TGS profile, no fixed set of observations was removed from the dataset. Results from both pipelines were compared to evaluate the robustness of the findings.

Associations between TGS profiles and biochemical markers were initially explored using Pearson correlation coefficients. As these analyses did not account for repeated measurements within subjects, they were considered exploratory. Longitudinal associations were subsequently evaluated using linear mixed-effects models including time, TGS profile, and their interaction as fixed effects, together with a random intercept for each participant. Models were fitted only when minimum data requirements were met (≥12 observations, ≥5 subjects, ≥2 time points, and ≥2 TGS levels). The theoretical maximum number of observations per biomarker was 240 (40 players × 6 assessments). Differences in available observations across biomarkers were attributable to occasional missing measurements and, in sensitivity analyses, the exclusion of biomarker-specific outliers identified using the IQR criterion.

For mixed-effects models, *p* values corresponding to TGS main effects and time × TGS interactions were adjusted using the Benjamini–Hochberg false discovery rate (FDR) procedure (*p*_adj_BH). Associations with *p*_adj_BH < 0.050 were considered statistically significant, whereas nominal associations failing FDR correction were interpreted as exploratory. Given the limited availability of elite professional football players, a convenience sample comprising all eligible athletes from the participating club was included.

Given the limited availability of elite professional football players, a convenience sample consisting of all eligible athletes from the participating club was used. Although no a priori sample size calculation was performed, post hoc power considerations indicated that the study was adequately powered to detect moderate-to-large correlation effects (r ≈ 0.40–0.50) at an α level of 0.050, whereas smaller associations may have remained undetected. Consequently, the analyses should be considered exploratory and hypothesis-generating.

Statistical significance was set at *p* < 0.050 for all analysis.

## 3. Results

Significant deviations from HWE were observed for *HFE* rs1799945 (*p* = 0.028) and *PGC1α* rs8192678 (*p* < 0.001). *CYP2D6* rs3892097 was monomorphic in the study population (MAF = 0%) and therefore HWE testing was not applicable, whereas all remaining variants showed no evidence of deviation (Table 3).

Sensitivity analyses comparing the primary analytical pipeline with analyses excluding dynamically identified outliers yielded comparable overall patterns of association. Therefore, the reported findings were not dependent on a limited number of extreme observations.

### 3.1. Multidimensional Correlation Mapping of Genetic and Biochemical Interactions

**Figure 1:**
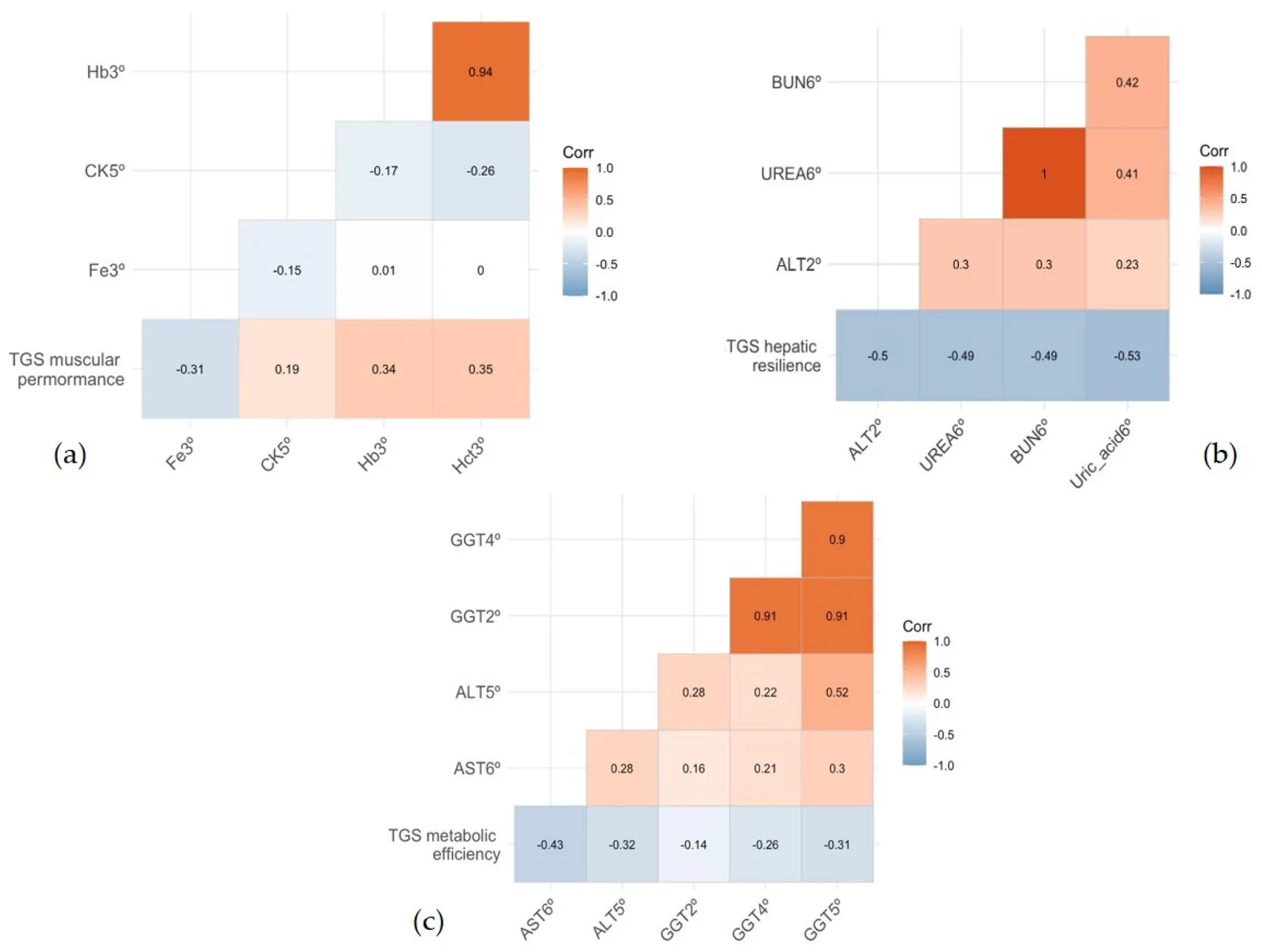
Correlation heatmaps illustrating the relationships between polygenic profiles (TGS) and selected biochemical markers across different seasonal time points. (**a**) Muscle performance profile showing associations with Fe, CK, Hb, and Hct; (**b**) hepatic resilience profile showing associations with ALT, urea, BUN, and uric acid; (**c**) metabolic efficiency profile showing associations with AST, ALT and GGT. Color gradients represent Pearson’s correlation coefficients (r), ranging from negative (blue) to positive (red) associations.

To provide an integrated overview of these associations, correlation patterns between polygenic profiles and key biochemical markers are summarized in Table 4 and Figure 1. Across all profiles, TGS values demonstrated consistent relationships with multiple biomarkers, with inverse correlations predominating for hepatic and metabolic markers, whereas positive associations were observed for selected indicators of muscle function and oxygen transport. These results further support the presence of coordinated profile-specific biochemical adaptations throughout the competitive season.

### 3.2. Muscle Performance Profile

Significant associations were observed between the muscle performance TGS and several biomarkers, including Fe, CK, Hb, and Hct (Table 4, Figure 1a). An inverse correlation was identified between muscle performance TGS and Fe at the 3rd biochemical assessment (r = −0.36, *p* = 0.017). In contrast, positive correlations were observed for CK at the 5th assessment (r = 0.32, *p* = 0.041), Hb at the 3rd assessment (r = 0.29, *p* = 0.046), and Hct at the 3rd assessment (r = 0.33, *p* = 0.024). These findings indicate that higher muscle performance polygenic scores were associated with distinct biochemical patterns across the competitive season, particularly in biomarkers related to muscle metabolism and oxygen transport.

### 3.3. Hepatic Resilience Profile

The hepatic resilience profile showed significant associations with several biomarkers related to hepatic function and protein metabolism, including ALT, urea, BUN, and uric acid (Table 4, Figure 1b). Negative correlations were observed between hepatic resilience TGS and ALT at the 2nd biochemical assessment (r = −0.39, *p* = 0.012), urea at the sixth assessment (r = −0.51, *p* = 0.011), and BUN at the 6th assessment (r = −0.51, *p* = 0.011). Uric acid also exhibited an inverse association with hepatic resilience TGS (r = −0.48), although this relationship did not reach statistical significance (*p* = 0.155) (Table 4). Overall, higher hepatic resilience polygenic scores were associated with lower values of several biomarkers related to hepatic function and nitrogen metabolism.

### 3.4. Metabolic Efficiency Profile

The metabolic efficiency profile showed significant associations with biomarkers related to hepatic function, including AST, ALT, and GGT (Table 4, Figure 1c). Negative correlations were observed between metabolic efficiency TGS and AST at the 6th biochemical assessment (r = −0.43, *p* = 0.044), ALT at the 5th assessment (r = −0.33, *p* = 0.025), and GGT at the 2nd (r = −0.38, *p* = 0.013), 4th (r = −0.47, *p* = 0.001), and 5th (r = −0.45, *p* = 0.002) biochemical assessments. Overall, higher metabolic efficiency TGS values were associated with lower levels of hepatic enzymes at several time points across the competitive season.

### 3.5. Linear Mixed-Effects Models

To account for the repeated-measures design of the study and the within-subject correlation arising from multiple biochemical assessments across the competitive season, linear mixed-effects models were fitted for all biomarker–TGS combinations meeting predefined data requirements. Models included time, TGS profile, and the time × TGS interaction as fixed effects, while participant identification was incorporated as a random intercept to account for individual variability.

Several nominally significant associations were identified within the mixed-effects framework (Table 5). In the muscle performance profile, a significant positive main effect of TGS was observed CK (β = 3.599, *p* = 0.011), indicating that athletes with higher muscle-performance genetic scores tended to present higher CK concentrations across the season. In addition, a positive time × TGS interaction was identified for hemoglobin at the third assessment point (β = 0.009, *p* = 0.031), suggesting potential temporal differences in hematological adaptation according to genetic profile.

Within the hepatic resilience profile, a negative interaction between TGS and the sixth biochemical assessment was observed for uric acid (β = −0.008, *p* = 0.045), indicating lower uric acid concentrations among athletes with more favorable hepatic genetic profiles at the end of the competitive season.

For the metabolic efficiency profile, significant negative time × TGS interactions were detected for ferritin (β = −0.549, *p* = 0.038) and GGT (β = −0.077, *p* = 0.028) at the fifth assessment point. These findings suggest that athletes with higher metabolic efficiency scores may exhibit distinct seasonal patterns in markers related to iron metabolism and hepatic function.

However, after adjustment for multiple comparisons using the Benjamini–Hochberg false discovery rate procedure, none of the observed associations remained statistically significant (all *p*_adj_BH > 0.050). Therefore, although the mixed-effects analyses identified several biologically plausible signals consistent with the exploratory correlation analyses, the evidence was insufficient to support robust longitudinal associations between polygenic profiles and biochemical markers after controlling multiple testing. Consequently, these findings should be interpreted as exploratory and hypothesis-generating rather than confirmatory. These mixed-effects analyses therefore represent the primary inferential framework for the longitudinal interpretation of the present dataset. Importantly, the application of FDR correction substantially reduced the number of statistically significant findings, suggesting that some associations identified in the exploratory correlation analyses may represent false-positive results arising from multiple testing.

## 4. Discussion

We hypothesized that professional football players with more favorable muscle, hepatic, and metabolic polygenic profiles would exhibit distinct longitudinal biochemical adaptations across the competitive season, particularly in biomarkers related to iron metabolism, muscle turnover, oxygen transport, and hepatic function. An important consideration is that the statistically significant associations identified in the correlation analyses were not consistently supported by the longitudinal mixed-effects modeling framework. Although several biomarkers demonstrated nominal associations with the different TGS profiles, none remained significant after adjustment for multiple comparisons. This observation suggests that some of the associations identified in the exploratory correlation analyses may reflect false-positive findings arising from the large number of statistical comparisons performed. Therefore, the present results should not be interpreted as definitive evidence of longitudinal genotype-dependent biochemical adaptations. Rather, they should be considered preliminary signals suggesting potential biological relationships that warrant confirmation in larger prospective cohorts specifically powered by repeated-measures genetic association analyses.

Our findings suggest that genetic predisposition may contribute to inter-individual variability in physiological responses to evolving training and competition demands, thereby reinforcing the need for precision-based approaches in sports medicine. The inverse association observed between muscle performance TGS and circulating iron—particularly evident at the third biochemical assessment—may indicate increased iron demand or turnover among players with favorable muscular genotypes. Iron plays a critical role in oxygen transport and energy metabolism as a key component of hemoglobin and myoglobin in skeletal muscle. Previous research has consistently shown that both overt and subclinical iron deficiency impair aerobic capacity and increase fatigue in athletes [1,43]. The observed pattern may therefore reflect enhanced cellular utilization in genetically advantaged individuals during periods of cumulative load, such as midseason.

Conversely, muscle performance TGS showed a positive association with CK, Hb, and Hct concentrations, indicating that athletes with a higher genetic predisposition for muscular performance tend to exhibit elevated levels of biomarkers related to muscle function and oxygen transport. Although increases in circulating CK are traditionally interpreted as markers of exercise-induced muscle damage, particularly in elite team-sport contexts, accumulating evidence suggests that higher CK activity may also reflect greater muscle mass, increased exposure to eccentric loading, higher cumulative training demands, and enhanced neuromuscular output rather than pathological processes [3,30,31,44,45,46,47]. Moreover, CK values in athletes are highly sport-specific and can substantially exceed reference values observed in the general population, reinforcing the importance of individualized interpretation within the context of training load, recovery status, and athletic characteristics [3,30,31,44,45].

Similarly, the positive associations observed between muscle TGS and Hb- and Hct-related parameters are physiologically consistent with the central role of oxygen transport capacity in athletic performance [48,49]. Therefore, the observed associations should be interpreted as reflecting inter-individual variability in physiological adaptation rather than evidence of maladaptive responses or increased injury susceptibility [4,50]. Potential biological explanations may involve differences in muscle structure, exercise tolerance, oxidative stress management, and recovery-related pathways influenced by exercise-responsive genetic variants; however, the observational nature of the present study precludes causal inference, and these mechanisms require confirmation through larger and mechanistic investigations.

An important consideration is that the muscle performance TGS evaluated in the present study was derived from genetic variants previously associated with muscle-related physiological traits and athletic performance phenotypes. However, direct measures of muscle performance, such as maximal strength, power output, torque production, sprint performance, or running speed, were not assessed. Therefore, the observed associations should be interpreted as relationships between a muscle-oriented genetic profile and biochemical adaptations rather than as direct evidence of enhanced athletic performance. Future investigations integrating both genetic profiles and objective performance measures will be necessary to determine the functional significance of these associations.

Extending this integrative physiological framework, our results indicate that a higher hepatic TGS is consistently associated with lower circulating levels of ALT, urea, and BUN throughout the competitive season, suggesting a potentially more favorable biochemical profile related to liver function and protein metabolism. Athletes with favorable hepatic polygenic profiles exhibited systematically reduced hepatic enzyme activity and nitrogenous waste accumulation, particularly at the 2nd and 6th biochemical assessments. This pattern is indicative of improved metabolic resilience and more effective nitrogen turnover and clearance under the sustained physiological stress typical of elite football. From a mechanistic perspective, lower ALT and nitrogenous byproducts may reflect enhanced hepatic efficiency, optimize amino acid metabolism, and reduce metabolic strain during high training and match loads [44,51,52].

Notably, these findings align with previous research demonstrating that elite athletes present distinct polygenic profiles in genes involved in xenobiotic metabolism and antioxidant defense—such as CYP450 family members and glutathione transferases (e.g., *GSTP1*, *GSTM1*, *GSTT1*)—which contribute to improved detoxification capacity and systemic recovery during prolonged or repeated exercise exposure [30,45]. This supports the hypothesis that enhanced metabolic efficiency, potentially mediated by improved detoxification pathways, mitochondrial function, and nitrogen balance, may be associated with biochemical responses compatible with improved adaptation to accumulated training load, although causality cannot be inferred from the present data [3,31,46,48,49].

Collectively, these findings underscore the relevance of incorporating polygenic hepatic profiles into athlete monitoring frameworks, reinforcing emerging evidence from sports genomics that genetic background contributes to inter-individual variability in metabolic adaptation and recovery capacity [47,50].

The metabolic efficiency genetic profile was consistently associated with markers of hepatic function and metabolic stress across the competitive season. Athletes with higher metabolic TGS exhibited systematically lower circulating levels of AST, ALT, and GGT, suggesting a more favorable hepatic response and reduced susceptibility to exercise-induced metabolic strain. These findings are in line with previous evidence indicating that advantageous polygenic variants in genes involved in mitochondrial biogenesis, xenobiotic metabolism, and redox regulation—such as *PGC1α*, *CYP2D6*, and glutathione-related enzymes (*GSTM1*, *GSTT1*, *GSTP1*)—have been previously linked to physiological pathways potentially involved in metabolic adaptation and recovery and improved detoxification capacity under conditions of sustained physiological load [30,44].

From a mechanistic perspective, reduced hepatic enzyme activity in athletes with higher metabolic TGS may reflect more efficient substrate utilization, improved oxidative metabolism, and a lower degree of cellular stress induced by repeated high-intensity efforts. Indeed, *PGC1α* plays a central role in mitochondrial biogenesis and metabolic regulation during exercise adaptation, contributing to enhanced oxidative capacity and energy efficiency [44]. However, these associations should be interpreted with caution, as hepatic enzyme fluctuations in elite athletes are influenced by multiple factors, including training load, nutritional status, and individual physiological variability [3].

This pattern may indicate differences in biochemical adaptation between athletes with distinct genetic profiles, although the biological mechanisms underlying these associations remain uncertain to exercise-induced stress. Potential underlying mechanisms could include improved oxidative stress handling, more efficient mitochondrial function, and optimized nitrogen metabolism, all of which have been previously linked to exercise adaptation and recovery processes.

In addition, the mild inverse association observed between metabolic efficiency TGS and glucose variability at preseason time points may indicate greater metabolic flexibility and more stable substrate utilization during early phases of the competitive cycle. Such metabolic stability could support the maintenance of energy availability under high training loads, although the strength of this association was modest and its practical implications remain to be clarified.

An important methodological consideration relates to the use of a candidate gene approach. Although several genetic variants included in the present study have been repeatedly associated with exercise-related phenotypes, candidate gene studies in sports genomics have been criticized because many reported associations have shown limited reproducibility across independent cohorts [24,32,47]. The selected SNPs were not chosen arbitrarily but were incorporated based on their biological relevance to pathways involved in muscle function, energy metabolism, iron homeostasis, oxidative stress regulation, and hepatic detoxification, as well as their previous inclusion in polygenic profiles developed in elite athletes and professional football players [10,29,32,34,41]. Nevertheless, the TGS approach used herein should be viewed as a hypothesis-driven framework rather than as a comprehensive measure of genetic predisposition.

Furthermore, contemporary sports genomics is progressively moving toward genome-wide polygenic score methodologies derived from large-scale GWAS datasets. Such approaches capture the contribution of thousands of variants distributed across the genome and generally provide greater predictive power and reproducibility than traditional candidate gene models [4,26,33]. However, robust GWAS-derived polygenic scores for many sport-specific phenotypes remain limited due to the relatively small number of elite athletes available for genome-wide studies and the considerable heterogeneity of performance traits. Consequently, the present study adopted a biologically informed TGS strategy focused on genetic variants with established functional relevance to exercise adaptation. Future studies should integrate GWAS-derived polygenic scores, larger athlete cohorts, and multi-omics data to improve the robustness and translational utility of precision sports medicine models [4,25]. While GWAS-derived weighted PRS may represent the future direction of sports genomics, validated effect sizes for many football-specific performance and adaptation traits remain unavailable, making equal-weighted TGS approaches a pragmatic and commonly used alternative in candidate-gene studies [4,24].

Taken together, these results reinforce the concept that metabolic efficiency represents a relevant polygenic trait associated with inter-individual variability in biochemical responses to training, rather than a direct determinant of injury risk. Future research should aim to elucidate the biological pathways underlying these associations—particularly those related to mitochondrial function, redox balance, and metabolic regulation—and to validate these findings in larger and multi-season cohorts using integrative, multi-omics approaches.

The findings of this study have relevant implications for sports science and elite football practice; (i) polygenic profiling may be incorporated into preseason assessments to characterize inter-individual variability in metabolic efficiency and recovery-related traits, thereby supporting the implementation of more individualized training load and recovery strategies, and (ii) the longitudinal monitoring of key biochemical markers—interpreted in conjunction with genetic predisposition—may provide an additional layer of information for identifying early signs of maladaptation, fatigue, or metabolic strain. This integrative approach could contribute to improving decision-making processes aimed at minimizing the risk of overreaching and optimizing athlete management.

The present findings suggest that polygenic profiles may contribute to inter-individual variability in biochemical responses to training and competition. However, the observed associations do not support immediate implementation in athlete monitoring programs. Further validation in larger cohorts and prospective studies is required before these genetic profiles can be considered for practical applications in personalized training, recovery, or nutritional interventions. Importantly, the associations observed in the present study should be interpreted within the context of its observational design. Although several statistically significant relationships were identified between polygenic profiles and biochemical markers, the magnitude of these associations was generally modest to moderate. Consequently, these findings should be considered hypothesis-generating rather than evidence of causal biological mechanisms.

Despite these strengths, several limitations should be acknowledged: (i) This study included only male professional football players from a single elite cohort assessed during a competitive season. Consequently, the observed genotype–biomarker associations may not be generalizable to athletes from other sports, competitive levels, female athletes, or non-athletic populations. (ii) Physiological adaptations to training are influenced by complex gene–environment interactions, and potentially relevant factors such as nutritional intake, sleep quality, psychological stress, individualized training loads, and intercurrent health conditions were not systematically controlled, which may have introduced residual confounding. (iii) Although relevant biomarkers were assessed, additional markers related to inflammation, oxidative stress, immune function, endocrine regulation, and mitochondrial activity may provide a more comprehensive characterization of physiological adaptation and recovery. (iv) The observational design precludes causal inference, and the relatively small sample size may have limited statistical power, increasing the risk of both type I and type II errors. (v) The TGS methodology follows the original framework proposed by Williams and Folland [35] and remains widely used in candidate-gene sports genomics research; however, it assumes equal contributions of all included polymorphisms and may therefore oversimplify the genetic architecture of complex exercise-related traits. Future studies should evaluate whether GWAS-derived polygenic scores or weighted multi-locus approaches provide superior predictive. (vi) Although linear mixed-effects models were implemented to account for repeated measurements, the combination of a limited sample size and multiple biochemical outcomes reduced the power to detect genotype–time interactions after correction for multiple testing. Consequently, the absence of significant findings after FDR correction should not be interpreted as evidence of absence of biological effects. (vii) No direct performance-related outcomes (e.g., strength, power, sprint performance, or running speed) were collected; therefore, the relationship between the evaluated polygenic profiles and objective measures of athletic performance remains to be established. (viii) Significant deviations from Hardy–Weinberg equilibrium were observed for *HFE* rs1799945, *PGC1α* rs8192678, and *CYP2D6* rs3892097. These deviations may reflect the highly selected nature of the cohort, the limited sample size, or population-specific genetic characteristics; therefore, associations involving these variants should be interpreted cautiously and confirmed in larger independent cohorts.

## 5. Conclusions

This longitudinal study suggests that polygenic profiles may be associated with inter-individual variability in biochemical adaptations across a competitive season in professional football players. Muscle-performance polygenic profiles were associated with iron status, creatine kinase, hemoglobin, and hematocrit, whereas hepatic and metabolic profiles showed associations with liver enzymes, urea, and blood urea nitrogen. These findings should be interpreted within the specific context of elite professional football and require validation across different sporting disciplines, competitive levels, and demographic populations before broader generalization can be considered.

Although causality cannot be inferred, these findings support the hypothesis that genetic background contributes to the heterogeneity of physiological responses to training and competition. Future studies involving larger cohorts, GWAS-derived polygenic scores, and integrative multi-omics approaches are warranted to validate these associations and clarify the biological mechanisms underlying athlete adaptation.

## Figures and Tables

**Table 1:** Baseline characteristics of professional football players.

		Professional Football Player (*n* = 40)
	Age, mean (SD)	24.80 (4.75)
	Weight, mean (SD)	76.7 (6.57)
	Height, mean (SD)	181 (5.98)
	BMI, mean (SD)	23.39 (1.46)
	Goalkeepers, *n* (%)	4 (10.0%)
	Defenders, *n* (%)	12 (30.0%)
Position	Midfields, *n* (%)	13 (32.5%)
	Forwards, *n* (%)	11 (27.5%)

SD, standard deviation.

**Table 2:** Genotype distribution in professional football players.

Genetic Profile	Gene Symbol	Gene Name	Polymorphism	dbSNP	Score
Muscle performance	*AMPD1*	Adenosine Monophosphate Deaminase 1	c.34C>T	rs17602729	CC = 2
					CT = 1
					TT = 0
	*ACE*	Angiotensin-Converting Enzyme	I/D	rs4646994	DD = 2
					DI = 1
					II = 0
	*ACTN3*	Actinin Alpha 3	c.1729C>T	rs1815739	CC = 2
					CT = 1
					TT = 0
	*CKM*	Creatine Kinase Muscle	c.*800A>G	rs8111989	GG = 2
					GA = 1
					AA = 0
	*MLCK*	Myosin Light Chain Kinase	c.49C>T	rs2700352	CC = 2
					CT = 1
					TT = 0
		Myosin Light Chain Kinase	c.37885C>A	rs28497577	CC = 2
					CA = 1
					AA = 0
Metabolic efficiency	*HFE*	Homeostatic Iron Regulator	c.845G>A	rs1800562	AA = 2
					AG = 1
					GG = 0
		Homeostatic Iron Regulator	c.187C>G	rs1799945	GG = 2
					GC = 1
					CC = 0
	*PGC1 α*	Peroxisome Proliferator-Activated Receptor Gamma Coactivator 1-alpha	c.1444G>A	rs8192678	GG = 2
					GA = 1
					AA = 0
Hepatic resilience	*CYP2D6*	Cytochrome P450 2D6	c.506-1G>A	rs3892097	GG = 2
					GA = 1
					AA = 0
	*GSTP1*	Glutathione S-transferase Pi 1	c.313A>G	rs1695	AA = 2
					AG = 1
					GG = 0
	*GSTM*	Glutathione S Transferase Mu 1	++/+−/−−		++ = 2
					+− = 1
					−− = 0
	*GSTT*	Glutathione S Transferase Theta 1	++/+−/−−		++ = 2
					+− = 1
					−− = 0

“+” indicates gene presence and “−” indicates gene absence.

**Table 3:** Genomic location and minor allele frequency (MAF) of selected profiles.

Symbol	Gene	dbSNP	Genomic Location	MAF Football Players	MAF (IBS) *	HWE	FIS
*AMPD1*	Adenosine Monophosphate Deaminase 1	rs17602729	1p13.2	10% (T)	14% (T)	*p* = 0.698	0.47
*ACE*	Angiotensin-Converting Enzyme	rs4646994	17q23.3	66.2% (I)	42.1% (I)	*p* = 0.159	0.13
*ACTN3*	Actinin Alpha 3	rs1815739	11q13.2	43.7% (T)	43.9% (T)	*p* = 0.388	0.16
*CKM*	Creatine Kinase Muscle	rs8111989	19q13.32	33.8% (G)	26.5% (G)	*p* = 0.599	−0.37
*MLCK*	Myosin Light Chain Kinase	rs2700352	3q21.1	25% (T)	20.1% (T)	*p* = 0.799	−0.12
*MLCK*	Myosin Light Chain Kinase	rs28497577	3q21.1	32.3% (A)	10.3% (A)	*p* = 0.323	−0.75
*HFE*	Homeostatic Iron Regulator	rs1800562	6p22.2	10% (A)	4.2% (A)	*p* = 0.193	−1.38
*HFE*	Homeostatic Iron Regulator	rs1799945	6p22.2	10% (G)	25.2% (G)	*p* = 0.028	0.406
*PGC1 α*	Peroxisome Proliferator-Activated Receptor Gamma Coactivator 1-alpha	rs8192678	4p15.2	22.5% (A)	38.8% (A)	*p* < 0.001	0.53
*CYP2D6*	Cytochrome P450 2D6	rs3892097	22q13.2	0% (A)	14.5% (A)	N/A monomorphic	1.00
*GSTP1*	Glutathione S-transferase Pi 1	rs1695	11q13.2	29.4% (G)	36.4% (G)	*p* = 0.603	0.21

IBS, Iberian population in Spain * [41,42]; FIS, inbreeding coefficient; HWE, Hardy–Weinberg equilibrium; MAF, minor allele frequency; SNP, single-nucleotide polymorphism. The distribution of polymorphisms for *GSTM1* and *GSTT1* was investigated in the population; however, since these are presence or absence gene mutations rather than SNPs, they do not have associated rs identifiers and are not represented in Ensembl. Consequently, HWE analysis could not be performed for these variants. N/A, not applicable.

**Table 4:** Correlation coefficients (r) of polygenic profiles (TGS) for key biochemical markers across seasonal assessments in professional football players.

Genetic Profile	Biochemical Marker	Biochemistry	r	*p* Value
TGS muscle performance	Fe	3rd	−0.36	0.017
TGS muscle performance	CK	5th	0.32	0.041
TGS muscle performance	Hb	3rd	0.29	0.046
TGS muscle performance	Hct	3rd	0.33	0.024
TGS hepatic resilience	ALT	2nd	−0.39	0.012
TGS hepatic resilience	Urea	6th	−0.51	0.011
TGS hepatic resilience	BUN	6th	−0.51	0.011
TGS hepatic resilience	Uric acid	6th	−0.48	0.155
TGS metabolic efficiency	AST	6th	−0.43	0.044
TGS metabolic efficiency	ALT	5th	−0.33	0.025
TGS metabolic efficiency	GGT	2nd	−0.38	0.013
TGS metabolic efficiency	GGT	4th	−0.47	0.001
TGS metabolic efficiency	GGT	5th	−0.45	0.002

ALT, alanine aminotransferase; AST, aspartate aminotransferase; BUN, blood urea nitrogen; CK, creatine phosphokinase; Fe, free blood iron; Hb, hemoglobin; Hct, hematocrit; GGT, gamma-glutamyl transferase; r, correlation coefficient; TGS, total genomic score.

**Table 5:** Nominally significant fixed effects identified in linear mixed-effects models assessing longitudinal associations between TGS profiles and biochemical markers.

Genetic Profile	Biomarker	β Factor	95%CI Low	95%CI High	Std. Error	t Value	*p* Value	*p*_adj_BH
Muscle performance	CK	3.599	0.86	6.33	1.39	2.578	0.011	0.095
Muscle performance	Hb	0.009	0.00	0.01	0.01	2.161	0.031	0.847
Hepatic resilience	Uric acid	−0.008	−0.01	−0.00	0.00	−2.013	0.045	0.984
Metabolic efficiency	Ferritin	−0.549	−1.06	−0.03	0.26	−2.082	0.038	0.623
Metabolic efficiency	GGT	−0.077	−0.14	−0.01	0.03	−2.209	0.028	0.623

CK, creatine kinase; Hb, hemoglobin; GGT, gamma-glutamyl transferase.

## Data Availability

The original contributions presented in this study are included in the article further inquiries can be directed to the corresponding author.
